# Applications of Large Language Models in Ovarian Cancer Management: Protocol for a Systematic Review and Meta-Analysis

**DOI:** 10.2196/88163

**Published:** 2026-07-10

**Authors:** Yanhong Wang, Jialiang Yao, Jianhui Tian, Yan Wang, Yun Yang

**Affiliations:** 1 Clinical Oncology Center, Shanghai Municipal Hospital of Traditional Chinese Medicine, Shanghai University of Traditional Chinese Medicine, No. 274 Zhijiang Middle Road, Jing’an District, Shanghai, 200071, China, 86 13761351319; 2 Institute of Oncology, Shanghai Municipal Hospital of Traditional Chinese Medicine, Shanghai University of Traditional Chinese Medicine, Shanghai, China; 3 Nursing Department, Shanghai Municipal Hospital of Traditional Chinese Medicine, Shanghai University of Traditional Chinese Medicine, Shanghai, China

**Keywords:** ovarian neoplasms, large language models, artificial intelligence, AI, systematic review, meta-analysis, Preferred Reporting Items for Systematic Reviews and Meta-Analyses, PRISMA

## Abstract

**Background:**

Ovarian cancer (OC) is a highly fatal gynecologic malignancy with complex management challenges and limited long-term survival for advanced stages. Large language models (LLMs)—including systems such as GPT-4, Claude, Google Gemini, and others—are emerging artificial intelligence (AI) tools capable of performing health care–related tasks such as diagnostic support, treatment planning, report generation, and patient communication. However, their applications in OC care have not yet been comprehensively assessed.

**Objective:**

This protocol outlines a systematic review and meta-analysis aimed at evaluating the use, performance, and clinical impact of LLMs in OC management. We will examine how LLMs have been applied across various domains (eg, diagnosis, prognosis, treatment planning, and patient engagement), the metrics used to assess their performance (eg, accuracy, sensitivity, and area under the curve), and their strengths and limitations.

**Methods:**

This review will be conducted in accordance with PRISMA-P (Preferred Reporting Items for Systematic Reviews and Meta-Analyses Protocols) guidelines. A comprehensive search strategy will be implemented across biomedical, technical, and Chinese-language databases (eg, PubMed, Embase, Web of Science, IEEE Xplore, and China National Knowledge Infrastructure) from inception to December 31, 2025. Eligible studies include clinical evaluations, validation studies, and real-world implementation reports involving LLMs in OC care. Two independent reviewers will perform screening, data extraction, and quality appraisal using validated tools (eg, version 2 of the Cochrane risk-of-bias tool for randomized trials, Risk of Bias in Nonrandomized Studies of Interventions, Quality Assessment of Diagnostic Accuracy Studies 2, and Prediction Model Study Risk of Bias Assessment Tool+AI). Outcomes of interest include model performance metrics, clinical process impacts, safety concerns, and usability. Meta-analyses will be conducted where feasible using random-effects models in R (*meta*, *metafor*, and *mada* packages), including bivariate models for sensitivity and specificity.

**Results:**

The review is currently in progress. The PROSPERO registration has been completed, and the literature search and selection process is underway. Study selection, data extraction, and quality assessment are expected to be completed by mid-2026. Final results will include pooled performance metrics (eg, accuracy, *F*_1_-score, and area under the curve), qualitative insights into clinical integration, and identification of limitations such as reporting bias or insufficient external validation.

**Conclusions:**

This systematic review will provide the first comprehensive synthesis of evidence on the application of LLMs in OC care. It will identify promising use cases, highlight safety and reporting challenges, and inform future research directions. The findings are expected to support evidence-based integration of LLMs into gynecologic oncology workflows while promoting transparency and methodological rigor in AI evaluation.

## Introduction

### Background

Ovarian cancer (OC) is a life-threatening gynecologic malignancy with a poor prognosis and high mortality rate [1]. It is the fifth most common cancer in women and the leading cause of gynecologic cancer–related death worldwide [2]. Despite advances in surgery and chemotherapy, the long-term survival for advanced OC remains low, highlighting an urgent need for innovations that can improve early detection, treatment decision-making, and overall management of this disease [3].

In recent years, the emergence of large language models (LLMs)—sophisticated artificial intelligence (AI) systems such as Claude, Google Gemini [4], Microsoft Copilot [5], Perplexity [6], DeepSeek [7], Llama [8], Ernie Bot, Kimi [9], and Grok—has opened up new possibilities in health care [10]. LLMs are capable of understanding and generating humanlike text, enabling applications such as answering medical questions, summarizing clinical reports, drafting consultation notes, and engaging in conversational interactions with patients or clinicians [11]. These models have rapidly been applied in various oncology contexts. For instance, studies on cancers such as lung, breast, and colorectal cancer have investigated the use of LLMs to assist with diagnosis, offer treatment recommendations, and educate patients, occasionally demonstrating performance comparable to that of human experts in specific tasks [12]. This trajectory aligns with broader frameworks mapping AI capabilities within smart health care ecosystems, where LLMs represent one component of an evolving suite of function-oriented clinical AI tools [13].

However, systematic evaluations reveal persistent gaps. Takita et al [14] conducted a systematic review and meta-analysis comparing generative AI models with physicians in diagnostic tasks across multiple medical specialties. They reported an overall diagnostic accuracy of 52.1% (95% CI, 47.0%-57.1%), representing the proportion of diagnostic responses judged correct according to the reference standards used in the included studies. Although the overall performance of generative AI models did not differ significantly from that of physicians as a whole, these models performed significantly worse than expert physicians. In oncology specifically, a systematic review covering 15 cancer types evaluated LLM applications across cancer decision-making tasks, including diagnostic support, treatment planning, summarization, translation, and communication of clinical information [15]. The review reported an average overall accuracy of 76.2% for LLM-assisted cancer decision-making tasks and a lower average accuracy of 67.4% for diagnostic tasks. These findings should be interpreted cautiously because the included studies varied in their clinical tasks, datasets, and evaluation criteria. Critically, recent meta-analytic work on explainable AI in clinical decision support emphasizes that robust evaluation must extend beyond technical metrics to encompass interpretability, usability, and user trust—dimensions increasingly recognized as essential for safe clinical integration [16].

In the context of OC specifically, the applications of LLMs are only beginning to be explored. A recent study by Piazza et al [17] compared ChatGPT’s independently generated responses to 8 clinical questions with the corresponding recommendations in the Italian Association of Medical Oncology guidelines. Although ChatGPT provided rapid responses, its answers were less clear, consistent, and comprehensive compared to the guideline-based recommendations.

The management of OC frequently demands intricate decision-making, such as interpreting genetic testing results, selecting among various treatment regimens, and handling disease recurrences—alongside substantial information processing—domains where LLMs could offer valuable assistance by synthesizing complex data and delivering evidence-based recommendations [18]. For example, a recent study evaluated ChatGPT’s performance in addressing clinical questions on OC management against National Comprehensive Cancer Network guidelines, revealing that, while the AI generated responses swiftly, they often fell short in matching the clarity and depth of expert-curated standards [19]. This underscores both the potential and current gaps in using LLMs for OC care. To date, no comprehensive systematic review has exclusively synthesized evidence on LLM applications and outcomes in OC management. Existing literature consists largely of broad multi-cancer reviews or narrative commentaries, leaving a critical knowledge gap for this high-mortality malignancy.

Recognizing the imperative for transparent and standardized reporting in medical AI, dedicated frameworks have emerged to guide scholarly communication. TRIPOD-AI (Transparent Reporting of a Multivariable Prediction Model for Individual Prognosis or Diagnosis–AI extension) and CONSORT-AI (Consolidated Standards of Reporting Trials–AI extension) provide structured criteria to enhance rigor in AI-based prediction model studies and clinical trials involving AI interventions, respectively [20,21]. Although this review focuses on synthesizing outcomes and applications rather than developing new models, we explicitly acknowledge these guidelines as vital context for appraising the methodological transparency and reporting quality of the included studies. During quality assessment, we will reference TRIPOD-AI and CONSORT-AI elements to evaluate adherence to emerging best practices in AI research reporting.

### Purpose of the Study

We aim to systematically collect and synthesize all available evidence on the use of LLMs in the management of OC. By doing so, we seek to understand the range of applications explored (from diagnosis and treatment planning to patient communication and beyond), evaluate the performance of LLMs in these applications (eg, accuracy and effectiveness), and identify the benefits and limitations observed. Ultimately, this review will inform clinicians, researchers, and policymakers about the current state of LLM integration in OC care and guide future research by highlighting areas of promise as well as areas needing improvement or further validation.

### Objectives

The primary objective of this review is to evaluate the applications and performance of LLMs in OC management. We will address the following key questions: (1) what tasks in OC care have LLMs been used for (eg, diagnostic support, treatment decision-making, prognosis prediction, clinical documentation, and patient education)? (2) What is the performance of LLMs on these tasks, and how does it compare to standard care or expert benchmarks (using metrics such as accuracy, sensitivity, specificity, precision, recall, *F*_1_-score, and area under the curve [AUC]; if sufficient data exist, we will pool these metrics across studies)? (3) What are the outcomes or impacts of using LLMs in OC management (including effects on clinical workflow, clinician decision-making, patient understanding or satisfaction, and any reported health outcomes)? (4) What challenges or limitations have been reported when applying LLMs to OC (eg, instances of diagnostic errors or AI “hallucinations,” failure of model outputs to align with clinical guidelines, or other safety and reliability concerns)? (5) How do different factors influence LLM performance in this domain (eg, whether performance varies by the type or version of LLM used, the specific clinical task or setting, or study and model design features)?

Ultimately, our review will help determine whether LLMs are ready for integration into OC care, identify the capacities in which they might be most useful, and outline what precautions or further developments are needed. If sufficient homogeneous data are available, we will perform meta-analyses to provide overarching estimates of LLM performance in this field.

## Methods

### Overview

This systematic review and meta-analysis protocol was developed in accordance with the PRISMA-P (Preferred Reporting Items for Systematic Reviews and Meta-Analyses Protocols) guidelines, follows the methodological standards recommended by the *Cochrane Handbook for Systematic Reviews of Interventions*, and has been prospectively registered in PROSPERO [22-26]. A comprehensive literature search will be conducted across biomedical and technical databases from inception to December 31, 2025. The full PRISMA-P checklist is provided in Checklist 1.

### Study Registration

This protocol has been registered in PROSPERO under registration number CRD420251144051. Any amendments to the protocol will be documented with the date and rationale and will be updated in the PROSPERO record. Any deviations from the original protocol will be transparently reported and justified in the final review.

### Primary Research Question

The primary research question is as follows: what is the scope and effectiveness of LLM applications in OC management? Specifically, to what extent have LLMs been evaluated for tasks such as diagnosis, treatment decision-making, prognosis prediction, clinical documentation, patient communication, and care coordination, and what performance or clinical process outcomes have been reported?

### Secondary Research Questions

The secondary research questions are as follows: do the reported effectiveness and clinical performance of LLMs vary by model type, clinical task, or input modality? Are there differences in performance according to study design, deployment setting, or language and data source? How do patient-, disease-, or system-level factors influence the observed utility of LLM-based applications in OC care?

### Information Sources

We will search MEDLINE (via PubMed), Embase (via Ovid), Web of Science Core Collection, Scopus, Cochrane Library, IEEE Xplore, ACM Digital Library, China National Knowledge Infrastructure, and Wanfang Data from database inception to December 31, 2025. These databases were selected to ensure broad coverage of biomedical, clinical, and computer science literature relevant to OC and LLMs. In addition, we will search Google Scholar for gray literature, conduct backward and forward citation searches of included studies, and check ClinicalTrials.gov and the World Health Organization International Clinical Trials Registry Platform for ongoing or unpublished studies. The particular search strategy is available in Textbox 1. All search activities, including search strings, date ranges, and number of retrieved records, will be documented in detail.

Textbox 1. **Search strategy for the PubMed database.**

- Search string 1: (“ovarian cancer”[All Fields] OR “ovarian carcinoma”[All Fields] OR “ovarian neoplasm”[All Fields] OR “ovarian neoplasms”[MeSH Terms] OR “ovarian tumor”[AllFields] OR “ovarian malignancy”[All Fields])
- Search string 2: (“large language model”[All Fields] OR “LLM”[All Fields] OR “ChatGPT”[AllFields] OR “GPT-3”[All Fields] OR “GPT-4”[All Fields] OR “Claude”[All Fields] OR”Gemini”[All Fields] OR “PaLM”[All Fields] OR “LLaMA”[All Fields] OR “Generative Pre-trained Transformer”[All Fields] OR “conversational AI”[All Fields] OR “generative AI”[All Fields])
- Search string 3: (“natural language processing”[MeSH Terms] AND (“generative model”[All Fields]OR “language generation”[All Fields] OR “instruction-tuned”[All Fields]))
- Search string 4: search strings 2 OR 3
- Search string 5: search strings 1 AND 4

### Search Strategy

The search strategy was developed iteratively. An initial scoping search in PubMed and Google Scholar was performed to identify relevant keywords and controlled vocabulary terms related to OC and generative LLMs. These terms were then combined using Boolean operators and adapted for each database according to platform-specific syntax and indexing rules. No study design restrictions will be applied during the search to maximize sensitivity.

The final search strategy was developed in PubMed and translated to the other databases. The full detailed search methodology for PubMed can be found in Multimedia Appendix 1, and the search terms will be adapted for application in other bibliographic databases. Immediately before final analysis, we will conduct an updated search to capture newly published studies.

### Study Selection

All identified references will be imported into a reference management software, and duplicates will be removed using automated detection followed by manual verification. Study selection will be conducted in 2 stages by 2 independent reviewers, with a third reviewer available to resolve disagreements.

In stage 1, titles and abstracts will be screened against the predefined eligibility criteria using an inclusive approach such that records that appear potentially relevant or on which uncertainty remains will proceed to full-text review. In stage 2, full texts of eligible or potentially eligible records will be independently assessed by 2 reviewers. Reasons for exclusion at the full-text stage will be documented. The study selection process will be summarized in a PRISMA (Preferred Reporting Items for Systematic Reviews and Meta-Analyses) flow diagram.

### Eligibility Criteria

We defined the eligibility criteria to align with the review objectives and ensure transparent and methodologically appropriate inclusion of studies evaluating LLMs in OC management. To improve readability, the inclusion and exclusion criteria are summarized in a structured double-column table (Table 1). Because this review is expected to include multiple study designs, we also prespecified design-specific appraisal and synthesis strategies.

**Table 1.** Inclusion and exclusion criteria for studies on large language models (LLMs) in ovarian cancer (OC) management.

Domain	Inclusion criteria	Exclusion criteria
Study design	RCTs^a^, quasi-experimental studies, nonrandomized interventional studies, prospective or retrospective cohort studies, case-control studies, diagnostic accuracy studies, model development or validation studies, cross-sectional surveys, real-world implementation reports, and qualitative studies relevant to LLM use in OC	Review articles, systematic reviews, meta-analyses, editorials, commentaries, letters, opinion papers, single case reports, studies without full text, and studies not focused on OC or without extractable OC-specific data from multi-cancer studies
Participants	Studies involving patients with OC at any stage of care, including screening, diagnosis, treatment decision-making, follow-up, survivorship, or palliative care; multi-cancer studies will be eligible only if OC-specific findings can be independently extracted	Studies on non-OC populations only or multi-cancer studies without separately extractable OC-specific results
Intervention	Application of transformer-based generative LLMs in OC-related tasks, such as diagnosis, treatment planning, prognostic assessment, documentation, report generation, communication between patients and health care professionals, literature or data synthesis, and patient education; eligible models include GPT^b^-series models, PaLM and Gemini-type models, Claude, Llama-based chat or instruction-tuned models, and other comparable generative systems	Studies evaluating rule-based systems, conventional machine learning tools, or nongenerative or encoder-only NLP^c^ models that do not meet this review’s operational definition of generative LLMs
Comparator	Comparator not required for eligibility; when available, comparators may include human experts, clinical guidelines, standard care, or other AI^d^ models	Studies lacking an interpretable evaluation context may still be included for narrative synthesis but not for meta-analysis if no meaningful reference standard is available
Outcome	At least one relevant outcome must be reported, including model performance metrics (eg, accuracy, sensitivity, specificity, precision, recall, *F*_1_-score, or AUC^e^) or clinical or operational outcomes (eg, decision-making impact, workflow efficiency, usability, satisfaction, safety, or information quality)	Studies without extractable performance, clinical, operational, or usability-related outcomes
Language	Articles published in English or Chinese	Articles published in languages other than English or Chinese

a RCT: randomized controlled trial.

b GPT: generative pretrained transformer.

c NLP: natural language processing.

d AI: artificial intelligence.

e AUC: area under the curve.

### Analytic Strategy by Study Design

Because the review includes heterogeneous forms of evidence, we prespecified a design-specific analytic plan. Randomized controlled trials will be appraised using version 2 of the Cochrane risk-of-bias tool for randomized trials (RoB 2) and synthesized quantitatively when outcome measures are sufficiently comparable. Quasi-experimental and nonrandomized intervention studies will be assessed using the Risk of Bias in Nonrandomized Studies of Interventions (ROBINS-I) and analyzed separately from randomized studies. Diagnostic accuracy studies will be appraised using the QUADAS-2 (Quality Assessment of Diagnostic Accuracy Studies 2) and, where at least 4 sufficiently comparable studies are available, pooled using a bivariate random-effects meta-analysis of sensitivity and specificity. Model development or validation studies will be evaluated using PROBAST+AI and synthesized quantitatively only when task type, outcome definitions, and evaluation frameworks are sufficiently aligned. Cross-sectional surveys and real-world implementation studies will generally be synthesized narratively unless comparable quantitative data permit pooling. Qualitative studies will be included only for narrative synthesis, focusing on user experience, acceptability, implementation barriers, and perceived utility.

To preserve interpretability, we will not directly pool results across fundamentally different study designs, clinical tasks, or reference standards. Instead, synthesis will be stratified by methodological type and task domain, and narrative synthesis will be used whenever statistical pooling is inappropriate.

### Types of Outcome Measures

#### Primary Outcomes

The primary outcomes are quantitative measures reflecting the diagnostic or predictive performance of LLMs in OC management. These include accuracy, sensitivity, specificity, precision, recall, *F*_1_-score, and AUC.

#### Secondary Outcomes

Secondary outcomes include broader clinical, operational, and implementation-related outcomes, such as changes in clinical decision-making, efficiency, user satisfaction, perceived usability, safety, information quality, and qualitative findings regarding implementation barriers or user experience. If quantitative pooling is not appropriate, these outcomes will be synthesized narratively and summarized in structured evidence tables.

### Data Abstraction and Extraction

A standardized data extraction form will be developed a priori and pilot-tested by 2 reviewers on a sample of included studies to ensure clarity, completeness, and consistency [27]. The core variables to be extracted are summarized in Textbox 2, which presents the data extraction framework for this review. After refinement, one reviewer will extract the data from all included studies, and a second reviewer will verify a subset for quality assurance.

Extracted information will include study characteristics, participant characteristics, details of the LLM intervention, comparator or reference standard when applicable, and all relevant outcomes. For dichotomous outcomes, we will extract event counts and sample sizes. For continuous outcomes, we will extract means, SDs, or other available summary statistics. For diagnostic studies, we will extract or derive true positives, false positives, true negatives, and false negatives whenever possible. If required information is unclear or missing, we will contact the study authors.

To avoid unit-of-analysis errors, we will extract at most one effect size per study per outcome for the main meta-analysis. If multiple LLM models or versions are reported within the same study using the same dataset, we will select the primary model designated by the authors. If no primary model is specified, we will select the model with the best performance on the study’s main outcome. Additional models will be considered only in subgroup or sensitivity analyses.

Textbox 2. **Data extraction framework for the included studies.**

**Study identification**

- First author
- Year of publication
- Country or region
- Journal or conference source

**Study characteristics**

- Study design
- Clinical or research setting
- Sample size
- Data source and recruitment approach

**Participant characteristics**

- Population type
- Ovarian cancer (OC)–related clinical stage or care context
- Inclusion of multi-cancer samples and whether OC-specific data were extractable

**Large language model characteristics**

- Name and version of the model
- Model category (general purpose or domain adapted)
- Deployment format (eg, stand-alone, application programming interface based, integrated system, or chatbot)
- Whether prompt engineering, fine-tuning, or external knowledge augmentation was used

**Task and application scenario**

- Clinical or research task addressed
- Input modality (text only or multimodal)
- Intended use (eg, diagnosis, treatment planning, prognosis, documentation, communication, or education)
- Comparator, if applicable

**Reference standard or comparator details**

- Human expert benchmark
- Clinical guideline or consensus statement
- Standard care pathway
- Other artificial intelligence model
- Diagnostic gold standard where applicable

**Outcomes extracted**

- Accuracy
- Sensitivity
- Specificity
- Precision
- Recall
- *F*_1_-score
- Area under the curve
- Time efficiency or workflow impact
- User satisfaction, usability, safety, or information quality
- Qualitative findings relevant to implementation or acceptability

**Data for quantitative synthesis**

- Raw diagnostic data if available (true positives, false positives, true negatives, and false negatives)
- Summary statistics and CIs
- Effect size data used for meta-analysis

**Risk of bias and reporting quality**

- Risk-of-bias assessment tool applied
- Key judgments from version 2 of the Cochrane risk-of-bias tool for randomized trials, Risk of Bias in Nonrandomized Studies of Interventions, Quality Assessment of Diagnostic Accuracy Studies 2, or Prediction Model Study Risk of Bias Assessment Tool+Artificial Intelligence
- Reporting transparency considerations relevant to the TRIPOD-AI (Transparent Reporting of a Multivariable Prediction Model for Individual Prognosis or Diagnosis–Artificial Intelligence extension) or CONSORT-AI (Consolidated Standards of Reporting Trials–Artificial Intelligence extension) where applicable

**Notes for synthesis**

- Eligibility for quantitative pooling
- Reason for narrative synthesis only if meta-analysis is not appropriate
- Additional methodological notes or reviewer comments

### Assessment of Study Quality

Two reviewers will independently assess risk of bias and methodological quality using design-specific tools. We will use the RoB 2 for randomized controlled trials, ROBINS-I for nonrandomized intervention studies, QUADAS-2 for diagnostic accuracy studies, and PROBAST+AI for prediction model studies. Disagreements will be resolved through discussion or consultation with a third reviewer.

We will summarize the risk-of-bias assessments in tables and figures and consider their impact in sensitivity and subgroup analyses. We will also assess the overall certainty of evidence for key outcomes using the GRADE (Grading of Recommendations Assessment, Development, and Evaluation) approach.

In addition, we will consider adherence to relevant AI-specific reporting frameworks such as TRIPOD-AI and CONSORT-AI as contextual references for evaluating transparency and reporting completeness.

### Measures of Treatment Effect

For comparative outcomes, dichotomous data will be summarized using risk ratios or odds ratios with 95% CIs, whereas continuous outcomes will be expressed as mean differences or standardized mean differences, as appropriate. Time-to-event outcomes, if available, will be summarized using hazard ratios with 95% CIs.

Because many included studies are expected to report model performance metrics rather than traditional treatment effects, we will also treat measures such as sensitivity, specificity, precision, recall, *F*_1_-score, accuracy, and AUC as effect measures for synthesis. Where necessary, these values will be calculated from reported raw data.

### Data Analysis and Effect Size Calculation

Meta-analyses will be conducted when a sufficient number of studies report comparable outcomes. In general, at least 2 studies will be required for a meta-analysis to be considered. Random-effects models will be used as the default approach.

For diagnostic accuracy outcomes, if at least 4 sufficiently comparable studies report both sensitivity and specificity, we will apply a bivariate random-effects model using the *mada* package in R (R Foundation for Statistical Computing) [28-30]. For other outcomes, such as accuracy, AUC, or *F*_1_-score, we will use random-effects meta-analysis using the *meta* or *metafor* packages when clinical tasks, datasets, and outcome definitions are sufficiently similar.

We will not pool outcomes across fundamentally different task domains. For example, diagnostic accuracy outcomes will not be combined with outcomes from patient communication or documentation tasks. When pooling is inappropriate because of insufficient data or substantial heterogeneity, results will be synthesized narratively.

### Assessment of Heterogeneity

Statistical heterogeneity will be assessed using the Cochran *Q* test and Higgins *I*^2^ statistic. Because the Cochran *Q* has low power when few studies are available, a significance threshold of *P*<.10 will be used. We will interpret *I*^2^ values approximately as follows: 25% or lower represents low heterogeneity, 25% to 50% represents moderate heterogeneity, and 75% or higher represents substantial heterogeneity.

If substantial heterogeneity is observed, we will explore plausible sources through subgroup analysis or meta-regression when feasible. If heterogeneity remains extreme and cannot be adequately explained, we may decide not to pool the results and, instead, provide a structured narrative synthesis. The between-study variance (τ^2^) will also be reported.

### Subgroup Analysis

If sufficient studies are available, prespecified subgroup analyses will be conducted according to key study or model characteristics, such as LLM type, clinical task, input modality, clinical context, study design, and risk-of-bias level. Where appropriate, tests for subgroup differences will be performed. If sufficient data are available, meta-regression may also be used to examine continuous moderators, such as year of publication or sample size.

### Sensitivity Analysis

Sensitivity analyses will be conducted to evaluate the robustness of the findings. These may include exclusion of outlier studies, exclusion of studies judged to be at high risk of bias, exclusion of very small studies, and comparison of fixed-effects and random-effects models when appropriate. For diagnostic accuracy outcomes, we may also compare joint bivariate models with separate univariate pooling approaches.

### Assessment of Reporting Bias

For meta-analyses including at least 10 studies, we will assess potential reporting bias using funnel plots and statistical tests such as the Egger regression test. Where appropriate, additional methods such as trim-and-fill analysis may be used. We will also consider selective outcome reporting during risk-of-bias assessment.

### Dealing With Missing Data

If relevant data are missing or incompletely reported, we will contact the corresponding authors for clarification. When possible, missing summary measures will be derived from available statistics using standard methods. If data remain unavailable, analyses will be based on the available data, and the potential impact of missing information will be considered in sensitivity analyses and discussed in the review.

### Ethical Considerations

Because this study is a systematic review and meta-analysis of published literature and does not involve individual patient data or direct participant contact, formal ethics approval is not required. The review will be conducted in accordance with accepted standards for research integrity, transparency, and responsible reporting.

## Results

**Figure 1.**
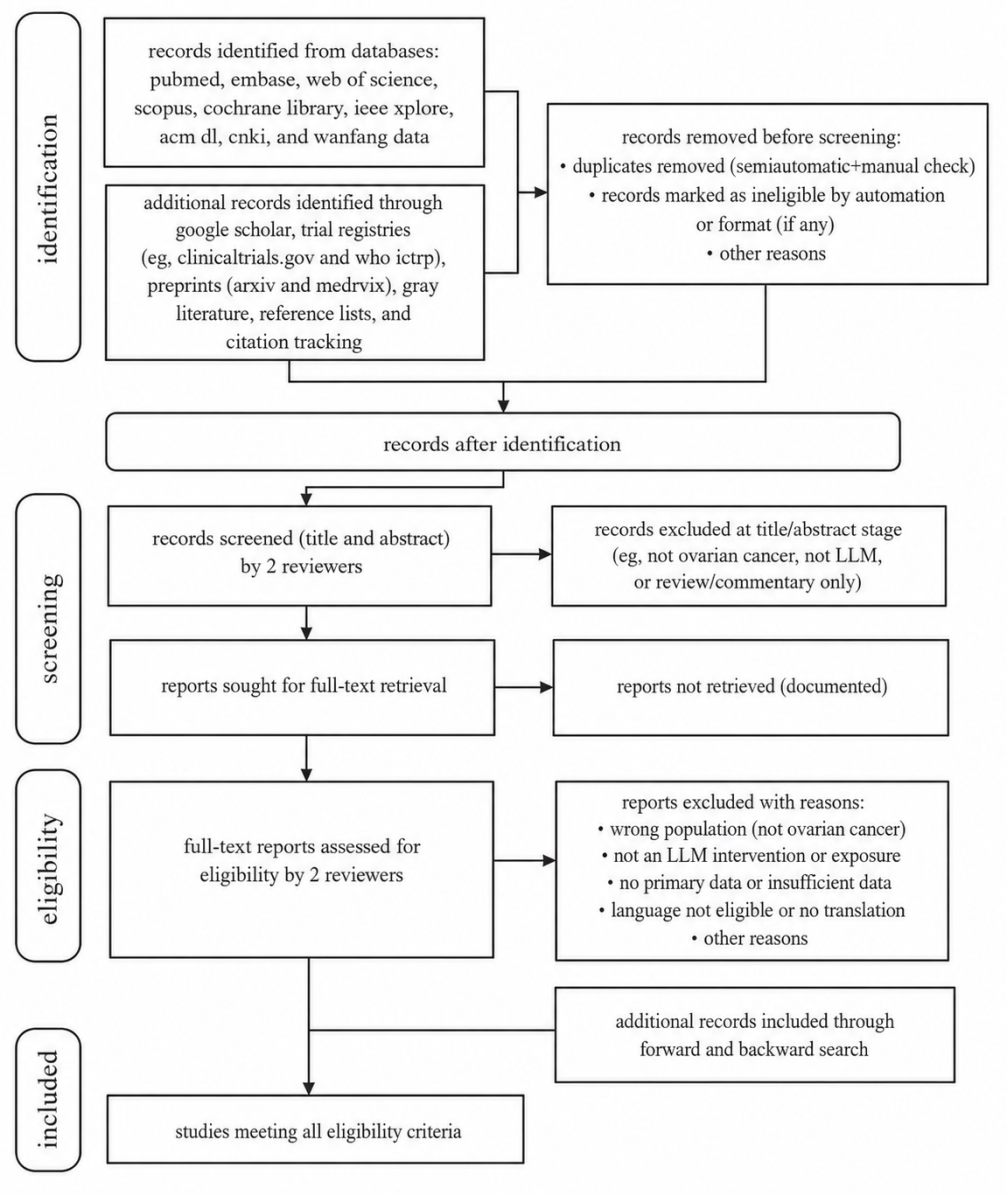
PRISMA (Preferred Reporting Items for Systematic Reviews and Meta-Analyses) flow diagram of the source of evidence selection process detailing records identified, screened, assessed for eligibility, excluded with reasons, and included in the review. CNKI: China National Knowledge Infrastructure; DL: Digital Library; ICTRP: International Clinical Trials Registry Platform; LLM: large language model; WHO: World Health Organization.

This study received funding in October 2025. The protocol was registered in PROSPERO (CRD420251144051), and the systematic review is currently in progress. At the time of manuscript submission, the database searches were still underway; therefore, the final number of retrieved records was not yet available. Study selection, data extraction, and quality assessment are expected to be completed by mid-2026. The completed systematic review and meta-analysis are anticipated to be published in Fall 2026. The final review will present pooled performance metrics, where appropriate, together with a narrative synthesis of the applications, clinical utility, safety considerations, and limitations of LLMs in OC management. The process of searching and selecting studies is depicted in a flow diagram in Figure 1. The findings of the review (including pooled performance metrics and qualitative syntheses of LLM applications) will be presented in the subsequent full review publication.

## Discussion

### Principal Anticipated Findings

This systematic review and meta-analysis is designed to evaluate how LLMs have been applied in OC management, including diagnosis, treatment decision support, prognostic assessment, clinical documentation, and patient communication. We anticipate that the review will identify domains where LLMs demonstrate promising utility—such as summarization or decision support—and areas where evidence is lacking or performance is inconsistent. Our hypothesis is that LLMs may achieve performance comparable to that of conventional methods in narrow tasks but face challenges in interpretability, safety, and generalizability in more complex clinical contexts.

We expect to observe substantial heterogeneity in model types, clinical use cases, and reported metrics. While some studies may report high accuracy or efficiency in patient-facing applications or documentation tasks, others may show limitations in tasks requiring nuanced clinical reasoning. Our findings may also reveal gaps in external validation and reporting consistency, making it difficult to generalize conclusions without careful subgroup analysis.

### Comparison to Prior Work

Existing reviews on AI in oncology often focus on imaging-based machine learning models or structured data analytics. Our protocol expands this by targeting natural language processing–based LLMs—particularly generative transformers—which remain underexamined in systematic syntheses, especially in OC care. Additionally, by including both English- and Chinese-language literature and searching both biomedical and computer science databases, we aim to capture complementary clinical and technical perspectives.

### Strengths and Limitations

This protocol’s strengths include its comprehensive scope (covering multiple LLM tasks), inclusion of both English- and Chinese-language studies, dual independent screening, and use of validated quality appraisal tools (RoB 2, ROBINS-I, and PROBAST+AI). However, we anticipate limitations, including study heterogeneity, variability in task definitions, small sample sizes, and inconsistent metric reporting. Additionally, we acknowledge potential reporting bias, especially in emerging LLM studies where positive results may be overemphasized.

### Research Integrity and Reporting Bias Considerations

Recent research on the causes of retractions in biomedical literature highlights the urgent need for critical evaluation of study reliability as issues such as inadequate peer review, limited data transparency, and ethical lapses are increasingly relevant to the rapidly expanding field of AI [31]. Similarly, evidence from a 2025 study reveals a widespread tendency toward “spin” in published clinical trials—where overly favorable conclusions are drawn despite weak or inconclusive results [32]. These insights are highly relevant to our review as LLM evaluations may be susceptible to selective reporting or exaggerated claims. Accordingly, we will examine whether the included studies make conclusions that are appropriately supported by their data and will reflect these judgments in our risk-of-bias assessment and interpretation of results.

### Future Directions

On the basis of the outcomes of this review, we hope to guide future LLM development in OC care by identifying specific tasks or conditions where performance is strong, as well as areas where improvement is needed—such as domain-specific fine-tuning, human oversight, and real-world validation. Our findings may also inform the development of reporting standards for AI-based clinical research, encouraging more transparent and reliable publications.

### Dissemination Plan

We will disseminate the results of this review through publication in peer-reviewed journals and presentations at academic conferences related to oncology and digital health. In addition, we will engage with stakeholders—including gynecologic oncologists, medical AI developers, and policy organizations—via institutional repositories and professional networks to ensure that the findings are accessible and actionable.

### Conclusions

This protocol outlines a comprehensive strategy to assess the current landscape of LLM applications in OC management. By systematically evaluating performance, usability, and reliability, the review will provide an evidence base to inform clinical practice, model development, and future research. Our approach recognizes both the potential and the limitations of LLMs and contributes to the growing dialogue on responsible AI integration in health care.

## Supplementary material

10.2196/88163 Multimedia Appendix 1 Search results.

10.2196/88163 Checklist 1 PRISMA-P checklist.

## Abbreviations

AI: artificial intelligence
AUC: area under the curve
CONSORT-AI: Consolidated Standards of Reporting Trials–Artificial Intelligence extension
GRADE: Grading of Recommendations Assessment, Development, and Evaluation
LLM: large language model
OC: ovarian cancer
PRISMA: Preferred Reporting Items for Systematic Reviews and Meta-Analyses
PRISMA-P: Preferred Reporting Items for Systematic Reviews and Meta-Analyses Protocols
PROBAST+AI: Prediction Model Study Risk of Bias Assessment Tool+Artificial Intelligence
QUADAS-2: Quality Assessment of Diagnostic Accuracy Studies 2
RoB 2: version 2 of the Cochrane risk-of-bias tool for randomized trials
ROBINS-I: Risk of Bias in Nonrandomized Studies of Interventions
TRIPOD-AI: Transparent Reporting of a Multivariable Prediction Model for Individual Prognosis or Diagnosis–Artificial Intelligence extension
