# Supplementary material for: Applications of Large Language Models in Ovarian Cancer Management: Protocol for a Systematic Review and Meta-Analysis
Source: JMIR Res Protoc. 2026 Jul 10;15:e88163. doi: 10.2196/88163 (PMC13353909; doi:10.2196/88163)
Supplement: Multimedia Appendix 1 [file resprot-v15-e88163-s001.docx]

**Multimedia Appendix 2**:Search History Result

Database: PubMed

Date range: Database inception – 01/09/2025

| **Search number** | **Query** | **Search details** | **Results** |
| --- | --- | --- | --- |
| 1 | "ovarian neoplasms"[MeSH Terms] | "ovarian neoplasms"[MeSH Terms] | 102,410 |
| 2 | ("ovarian cancer"[All Fields] OR "ovarian carcinoma"[All Fields] OR "ovarian neoplasm"[All Fields] OR "ovarian tumor"[All Fields] OR "ovarian malignancy"[All Fields]) | ("ovarian cancer"[All Fields] OR "ovarian carcinoma"[All Fields] OR "ovarian neoplasm"[All Fields] OR "ovarian tumor"[All Fields] OR "ovarian malignancy"[All Fields]) | 91,297 |
| 3 | #1 OR #2 | ("ovarian neoplasms"[MeSH Terms] OR "ovarian cancer"[All Fields] OR "ovarian carcinoma"[All Fields] OR "ovarian neoplasm"[All Fields] OR "ovarian tumor"[All Fields] OR "ovarian malignancy"[All Fields]) | 131,792 |
| 4 | ("large language model"[All Fields] OR LLM[All Fields]) | ("large language model"[All Fields] OR LLM[All Fields]) | 5,389 |
| 5 | ("ChatGPT"[All Fields] OR "GPT-3"[All Fields] OR "GPT-4"[All Fields] OR "Generative Pre-trained Transformer"[All Fields]) | ("ChatGPT"[All Fields] OR "GPT-3"[All Fields] OR "GPT-4"[All Fields] OR "Generative Pre-trained Transformer"[All Fields]) | 9,605 |
| 6 | ("BERT"[All Fields] OR "T5"[All Fields] OR "LLaMA"[All Fields] OR "PaLM"[All Fields]) | ("BERT"[All Fields] OR "T5"[All Fields] OR "LLaMA"[All Fields] OR "PaLM"[All Fields]) | 48,658 |
| 7 | ("conversational AI"[All Fields] OR "dialogue model"[All Fields] OR "generative AI"[All Fields]) | ("conversational AI"[All Fields] OR "dialogue model"[All Fields] OR "generative AI"[All Fields]) | 3,146 |
| 8 | (#4 OR #5 OR #6 OR #7) | ("large language model"[All Fields] OR LLM[All Fields] OR "ChatGPT"[All Fields] OR "GPT-3"[All Fields] OR "GPT-4"[All Fields] OR "Generative Pre-trained Transformer"[All Fields] OR "BERT"[All Fields] OR "T5"[All Fields] OR "LLaMA"[All Fields] OR "PaLM"[All Fields] OR "conversational AI"[All Fields] OR "dialogue model"[All Fields] OR "generative AI"[All Fields]) | 62,680 |
| 9 | ("artificial intelligence"[MeSH Terms] OR "natural language processing"[MeSH Terms]) AND "language model"[All Fields] | ("artificial intelligence"[MeSH Terms] OR "natural language processing"[MeSH Terms]) AND "language model"[All Fields] | 2,055 |
| 10 (Final query) | (#3) AND (#8 OR #9) | ("ovarian neoplasms"[MeSH Terms] OR "ovarian cancer"[All Fields] OR "ovarian carcinoma"[All Fields] OR "ovarian neoplasm"[All Fields] OR "ovarian tumor"[All Fields] OR "ovarian malignancy"[All Fields]) AND ("large language model"[All Fields] OR LLM[All Fields] OR "ChatGPT"[All Fields] OR "GPT-3"[All Fields] OR "GPT-4"[All Fields] OR "Generative Pre-trained Transformer"[All Fields] OR "BERT"[All Fields] OR "T5"[All Fields] OR "LLaMA"[All Fields] OR "PaLM"[All Fields] OR "conversational AI"[All Fields] OR "dialogue model"[All Fields] OR "generative AI"[All Fields] OR ("artificial intelligence"[MeSH Terms] OR "natural language processing"[MeSH Terms]) AND "language model"[All Fields]) | 30 |
