# Supplementary material for: Applications of Large Language Models in Ovarian Cancer Management: Protocol for a Systematic Review and Meta-Analysis
Source: JMIR Res Protoc. 2026 Jul 10;15:e88163. doi: 10.2196/88163 (PMC13353909; doi:10.2196/88163)
Supplement: Checklist 1 [file resprot-v15-e88163-s002.docx]

# **Supplemental File 1:PRISMA-P Checklist for 'Applications of Large Language Models in Ovarian Cancer Management: A Protocol for Systematic Review and Meta-analysis'**

| Section and Topic | Item No | Checklist Item | Page number(s) |
| --- | --- | --- | --- |
| Administrative Information | 1a | Identify the report as a protocol of a systematic review | p.1 |
| Administrative Information | 1b | If the protocol is for an update of a previous systematic review, identify as such | — |
| Administrative Information | 2 | If registered, provide the name of the registry (such as PROSPERO) and registration number | p.2 |
| Administrative Information | 3a | Provide name, institutional affiliation, e-mail address of all protocol authors; provide physical mailing address of corresponding author | p.1 |
| Administrative Information | 3b | Describe contributions of protocol authors and identify the guarantor of the review | p.27 |
| Administrative Information | 4 | If the protocol represents an amendment of a previously completed or published protocol, identify as such and list changes; otherwise, state plan for documenting important protocol amendments | p.26 |
| Administrative Information | 5a | Indicate sources of financial or other support for the review | p.2 |
| Administrative Information | 5b | Provide name for the review funder and/or sponsor | p.2 |
| Administrative Information | 5c | Describe roles of funder(s), sponsor(s), and/or institution(s), if any, in developing the protocol | p.26 |
| Introduction | 6 | Describe the rationale for the review in the context of what is already known | p.3–6 |
| Introduction | 7 | Provide an explicit statement of the question(s) the review will address with reference to participants, interventions, comparators, and outcomes (PICO) | p.7–8 |
| Methods | 8 | Specify the study characteristics and report characteristics to be used as criteria for eligibility for the review | p.9–11 |
| Methods | 9 | Describe all intended information sources with planned dates of coverage | p.11–12 |
| Methods | 10 | Present draft of search strategy to be used for at least one electronic database | p.12–13, Table 3 |
| Methods | 11a | Describe the mechanism(s) that will be used to manage records and data throughout the review | p.13 |
| Methods | 11b | State the process that will be used for selecting studies through each phase of the review | p.13–14 |
| Methods | 11c | Describe planned method of extracting data from reports, any processes for obtaining and confirming data from investigators | p.15 |
| Methods | 12 | List and define all variables for which data will be sought, any pre-planned data assumptions and simplifications | p.24–25 |
| Methods | 13 | List and define all outcomes for which data will be sought, including prioritization of main and additional outcomes, with rationale | p.8, p.17–18 |
| Methods | 14 | Describe anticipated methods for assessing risk of bias of individual studies | p.15–16 |
| Methods | 15a | Describe criteria under which study data will be quantitatively synthesised | p.17–18 |
| Methods | 15b | If data are appropriate for quantitative synthesis, describe planned summary measures, methods of handling data and methods of combining data from studies | p.18–19 |
| Methods | 15c | Describe any proposed additional analyses (such as sensitivity or subgroup analyses, meta-regression) | p.19–20 |
| Methods | 15d | If quantitative synthesis is not appropriate, describe the type of summary planned | p.20–21 |
| Methods | 16 | Specify any planned assessment of meta-bias(es) | p.18–19 |
| Methods | 17 | Describe how the strength of the body of evidence will be assessed | p.19–21 |
